# PIF4–Mediated Activation of *YUCCA8* Expression Integrates Temperature into the Auxin Pathway in Regulating *Arabidopsis* Hypocotyl Growth

**DOI:** 10.1371/journal.pgen.1002594

**Published:** 2012-03-29

**Authors:** Jiaqiang Sun, Linlin Qi, Yanan Li, Jinfang Chu, Chuanyou Li

**Affiliations:** State Key Laboratory of Plant Genomics, National Centre for Plant Gene Research, Institute of Genetics and Developmental Biology, Chinese Academy of Sciences, Beijing, China; Peking University, China

## Abstract

Higher plants adapt their growth to high temperature by a dramatic change in plant architecture. It has been shown that the transcriptional regulator phytochrome-interacting factor 4 (PIF4) and the phytohormone auxin are involved in the regulation of high temperature–induced hypocotyl elongation in *Arabidopsis*. Here we report that PIF4 regulates high temperature–induced hypocotyl elongation through direct activation of the auxin biosynthetic gene *YUCCA8* (*YUC8*). We show that high temperature co-upregulates the transcript abundance of *PIF4* and *YUC8*. PIF4–dependency of high temperature–mediated induction of *YUC8* expression as well as auxin biosynthesis, together with the finding that overexpression of *PIF4* leads to increased expression of *YUC8* and elevated free IAA levels *in planta*, suggests a possibility that PIF4 directly activates *YUC8* expression. Indeed, gel shift and chromatin immunoprecipitation experiments demonstrate that PIF4 associates with the G-box–containing promoter region of *YUC8*. Transient expression assay in *Nicotiana benthamiana* leaves support that PIF4 directly activates *YUC8* expression *in vivo*. Significantly, we show that the *yuc8* mutation can largely suppress the long-hypocotyl phenotype of *PIF4*–overexpression plants and also can reduce high temperature–induced hypocotyl elongation. Genetic analyses reveal that the *shy2-2* mutation, which harbors a stabilized mutant form of the IAA3 protein and therefore is defective in high temperature–induced hypocotyl elongation, largely suppresses the long-hypocotyl phenotype of *PIF4*–overexpression plants. Taken together, our results illuminate a molecular framework by which the PIF4 transcriptional regulator integrates its action into the auxin pathway through activating the expression of specific auxin biosynthetic gene. These studies advance our understanding on the molecular mechanism underlying high temperature–induced adaptation in plant architecture.

## Author Summary

Exposure of *Arabidopsis* to high temperature (29°C) results in a dramatic hypocotyl elongation. The basic helix-loop-helix transcription factor PIF4 and the phytohormone auxin play essential roles in high temperature–mediated induction of *Arabidopsis* hypocotyl elongation. However, the possible molecular linkage between PIF4 and the auxin pathway in regulating high temperature–induced adaptative growth remains unknown. Here, we report that high temperature–induced elevation of *YUCCA8* (*YUC8*) transcripts and endogenous free IAA levels is dependent on the function of PIF4. In particular, we provide evidence that PIF4 directly activates the expression of *YUC8* to upregulate auxin biosynthesis, as a consequence, achieves high temperature–induced hypocotyl elongation. In addition, we found that SHY2/IAA3 is an important component of the PIF4–auxin pathway in regulating high temperature–induced hypocotyl elongation. Overall, our results establish a direct connection between the PIF4 transcription factor and the auxin pathway in regulating high temperature–induced adaptation growth.

## Introduction

Higher plants continually sense environmental conditions to adapt their growth and development. To a large extent, this is achieved through integrating environmental cues into the growth-regulating hormonal pathways. Exposure of *Arabidopsis thaliana* plants to high temperature (29°C) results in dramatic plant architecture changes including rapid hypocotyl elongation, leaf hyponasty, and early flowering [1]–[4]. High temperature-induced hypocotyl elongation of *Arabidopsis* plants provides an ideal model system to investigate the regulatory mechanisms underlying adaptive growth of plants to their ever-changing environments. Among the endogenous cues involved in the regulation of high temperature-induced hypocotyl elongation is the plant hormone auxin [3]. An early observation revealed a correlation between high temperature-induced hypocotyl elongation and high temperature-induced elevation of endogenous free indole-3-acetic acid (IAA) levels [3]. Genetic analyses found that high temperature-induced hypocotyl elongation is sharply reduced in *Arabidopsis* mutants defective in auxin biosynthesis, transport or signaling [3]. Together, these data attribute an essential role of the auxin pathway in mediating high temperature-induced hypocotyl elongation.

It is long-recognized that auxin has profound effects on plant growth and development. A combination of physiological, biochemical, pharmacological and molecular genetic studies provide an ever-growing body of insights on our understanding of the auxin biosynthesis pathway [5], [6]. It is generally believed that, IAA, the main auxin in higher plants, can be synthesized from tryptophan (Trp)-dependent and -independent pathways [5]. Among the best-characterized enzymes involved in the Trp-dependent auxin biosynthetic pathway are the YUCCA (YUC) family of flavin-containing monooxygenases [5], [7]–[9] and the TRYPTOPHAN AMINOTRANSFERASE OF ARABIDOPSIS1/TRYPTOPHAN AMINOTRANSFERASE-RELATED (TAA1/TAR) family of aminotransferases [5], [10], [11]. A wealth of genetic evidence indicated that, while inactivating members of the *YUC* family genes causes dramatic developmental defects [8], [9], overexpression of the *YUC* family genes leads to auxin overproduction and long hypocotyl phenotype in *Arabidopsis* [7]. Although mutation of *TAA1* or its close homologs (*TAR* genes) leads to developmental defects similar to those of the *yuc* mutants [10], [11], overexpression of TAA1/TAR does not cause obvious developmental phenotype, suggesting that TAA1/TAR probably do not catalyze a rate-limiting step in IAA biosynthesis [10], [11]. Interestingly, recent studies provide evidence that TAA1/TARs and YUCs may act in a common linear biosynthetic pathway for auxin production [6], [12], [13].

In addition to auxin, a family of phytochrome-interacting factors (PIFs), which encode basic helix-loop-helix (bHLH) transcription factors, have been shown to be central integrators of versatile environmental and hormonal signals during plant adaptive growth [14], [15]. Among the PIF family of transcriptional regulators, a selective function of PIF4 in high temperature-induced hypocotyl elongation has recently been reported [1], [16]. These studies revealed that high temperature induced a rapid elevation of *PIF4* transcript levels and that the *pif4* mutant largely lost the robust enhancement of hypocotyl elongation induced by high temperature [1].

In the context that both the transcription factor PIF4 and the phytohormone auxin are required for high temperature-induced hypocotyl elongation, a fascinating hypothesis is that PIF4 may directly link the auxin pathway in regulating plant adaptation growth to high temperature. We provide here evidence that, in response to high temperature, PIF4 directly activates *YUC8* expression and thus elevates endogenous free IAA levels. We also show that the SHY2/IAA3 protein is a downstream component of the PIF4-auxin signaling pathway in regulating high temperature-induced hypocotyl elongation. Our results exemplify how a transcriptional regulator integrates environmental cues with endogenous hormonal signaling to mediate specialized developmental changes in regulating plant adaptive growth.

## Results

### Loss of PIF4 Function Impairs High Temperature–Induced Elevation of *YUC8* Transcripts and Endogenous Free IAA Levels

**Figure 1:**
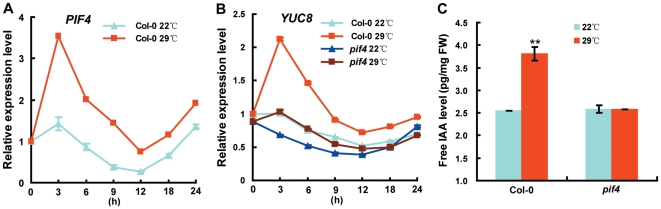
Loss of PIF4 Function Disrupts the High Temperature–Induced Elevation of *YUC8* Transcripts and Free IAA Levels. (A–B) High temperature-induced expression patterns of *PIF4* and *YUC8* in wild type (Col-0) or the *pif4* mutant. Six-d-old Col-0 and *pif4* seedlings grown at 22°C in continuous light were transferred to 29°C in continuous light or were continually placed at 22°C for a 24 h time course, respectively. The 22°C-grown and 29°C-grown seedlings for each time point were harvested at the same time for RNA extraction and qRT-PCR analyses. Transcript levels of target genes were normalized to the *ACTIN7* expression and were relative to those of untreated Col-0 seedlings (0 h). Data shown are average and SD of triplicate reactions. Shown are representative data from one biological replicate; three biological replicates were conducted, yielding similar results. (C) High temperature-induced elevation of free IAA levels in hypocotyls of Col-0 and *pif4*. The hypocotyls of 6-d-old wild-type and *pif4* mutant seedlings grown at 22°C and 29°C in continuous light, respectively, were harvested for free IAA measurement. Data shown are average±SD. Student's *t*-test between 22°C and 29°C grown plants for each genotype was performed (**, P<0.01). Shown are representative data from one biological replicate; This experiment was conducted for three biological replicates, yielding similar results.

It has been shown that high temperature activates the expression of the transcription factor PIF4 [1], and elevates endogenous free IAA levels [3] in *Arabidopsis*. To explore the possible molecular linkage between PIF4 and the auxin pathway in regulating high temperature-mediated adaptation growth, we examined high temperature-induced expression of *PIF4* and the *YUCCA* (*YUC*) family of auxin biosynthetic genes [5]. Consistent with previous reports [1], , when wild type (WT) seedlings grown at 22°C for 6 days were transferred to 29°C in continuous light over a 24 h time course, *PIF4* transcript abundance was transiently elevated to a peak level at 3 h after transfer (Figure 1A). Correlating with an increased expression of *PIF4*, high temperature also markedly increased transcript abundance of *YUC8* with a peak at 3 h in WT seedlings (Figure 1B). Closer observation with a narrower range of time points revealed that high temperature-mediated induction of *YUC8* expression occurred generally later than that of *PIF4* (Figure S1). Parallel experiments indicated that high temperature did not upregulate the expression of other *YUCCA* family genes tested (Figure S2). We then compared high temperature-induced *YUC8* expression between WT and the *pif4* mutant, which has been shown to be defective in high temperature-induced adaptations in plant architecture (Figure 1B). As shown in Figure 1B, the basal expression levels of *YUC8* were already low in *pif4* seedlings and, significantly, high temperature-induced upregulation of *YUC8* expression was largely abolished in this mutant, indicating that the function of PIF4 is important for the basal- and high temperature-induced expression of *YUC8*.

The *pif4* mutation impairs high temperature-induced upregulation of *YUC8* expression suggests that this mutation may also affect high temperature-induced elevation of free IAA levels. To test this, we compared high temperature-induced elevation of free IAA levels in WT and *pif4* seedlings. For these experiments, we grew seedlings at 22°C or 29°C in continuous light for 6 days and collected hypocotyls for IAA measurement. Consistent with a previous report [3], high temperature increased free IAA levels of WT seedlings by around 50% (Figure 1C). As expected, high temperature-induced elevation of free IAA levels was abolished in the *pif4* mutant (Figure 1C), indicating that PIF4 is also required for high temperature-induced elevation of auxin biosynthesis. Together, these results suggest that *PIF4* and *YUC8* may function in linking temperature and auxin pathway in regulating hypocotyl elongation.

### Overexpression of *PIF4* Upregulates *YUC8* Expression and Leads to Elevated Endogenous Free IAA Levels

**Figure 2:**
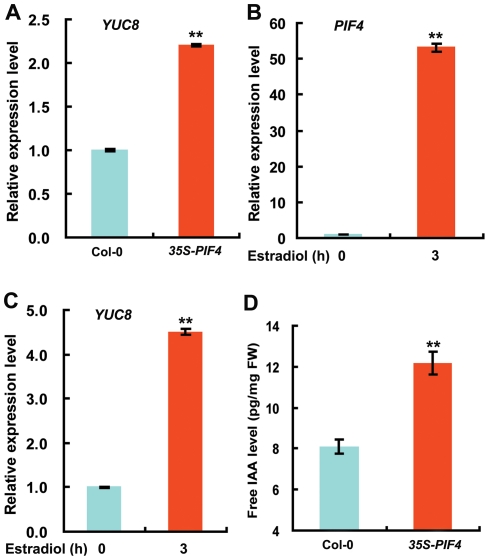
Overexpression of *PIF4* Increases the Expression of *YUC8* and Elevates Endogenous Free IAA Levels. (A) *YUC8* expression in wild type (Col-0) and *35S-PIF4* plants. Six-d-old Col-0 and *35S-PIF4* seedlings grown in normal growth conditions (22°C) were harvested at the same time for RNA extraction and qRT-PCR analyses. Transcript levels of *YUC8* were normalized to the *ACTIN7* expression and then were relative to those of Col-0 seedlings. Data shown are average and SD of triplicate reactions. Student's *t*-test between Col-0 and *35S-PIF4* seedlings was performed (**, P<0.01). Shown are representative data from one biological replicate; three biological replicates were conducted, yielding similar results. (B–C) *PIF4* and *YUC8* expression in transgenic plants containing a chemical-inducible construct *pMDC7:PIF4*. Eight-d-old *pMDC7:PIF4* seedlings were untreated or treated with 10 µM estradiol for 3 h before harvest for RNA extraction and qRT-PCR analyses. Transcript levels of target genes were normalized to the *ACTIN7* expression and then were relative to those of untreated seedlings (0 h). Data shown are average and SD of triplicate reactions. Student's *t*-test between estradiol-treated and untreated plants was performed (**, P<0.01). Shown are representative data from one biological replicate; three biological replicates were conducted, yielding similar results. (D) Overexpression of *PIF4* leads to increased free IAA levels. Eight-d-old seedlings of wild type and *35S-PIF4* seedlings grown in normal growth conditions (22°C) were harvested at the same time for free IAA measurement. Data shown are average±SD. Student's *t*-test between wild-type and *35S-PIF4* plants was performed (**, P<0.01). Shown are representative data from one biological replicate; this experiment was conducted for three biological replicates, yielding similar results.

As a first step to test the possibility that PIF4 may directly regulate *YUC8* expression during high temperature-induced adaptation growth, we examined *YUC8* expression in transgenic plants overexpressing *PIF4* (*35S-PIF4*). Like the reported *yucca* mutants which contain increased endogenous auxin levels [7], *35S-PIF4* plants show a long hypocotyl phenotype that resembles high temperature-grown WT seedlings (Figure S3). As revealed by quantitative reverse transcription-polymerase chain reaction (qRT-PCR) assays, the expression of *YUC8* (Figure 2A), but not that of *TAA1* (Figure S4), was substantially increased in *35S-PIF4* seedlings as compared to WT. We also generated *PIF4*-overexpression plants (*pMDC7:PIF4*) using the chemical inducible vector *pMDC7* [17]. In the presence of the chemical inducer estradiol, *pMDC7:PIF4* seedlings show increased expression of *PIF4* (Figure 2B) and display a long hypocotyl phenotype like *35S-PIF4* seedlings (Figure S5). As expected, *YUC8* expression was considerably elevated following estradiol induction (Figure 2C). Consistently, measurement of auxin revealed that the free IAA levels in *35S-PIF4* plants were increased by 50% as compared to those in WT plants (Figure 2D). In line with increased free IAA levels in *35S-PIF4* plants, the expression of the auxin responsive *DR5:GUS*, a widely used reporter of auxin response, was clearly enhanced in the basal region of *35S-PIF4* hypocotyls (Figure S6). These data together indicate that overexpression of *PIF4* leads to increased expression of the auxin biosynthetic gene *YUC8* and, as a result, elevated endogenous free IAA levels *in planta*.

### PIF4 Directly Binds to the Promoter Region of *YUC8*

**Figure 3:**
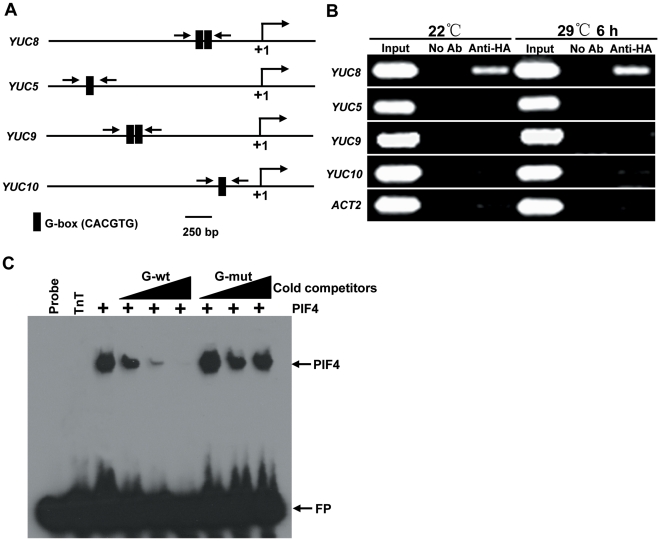
PIF4 Directly Binds to the Promoter Region of *YUC8*. (A) Illustration of the *YUC5*, *YUC8*, *YUC9* and *YUC10* promoter regions showing the presence of G-box DNA motifs. The arrows indicate positions of primers used for ChIP-PCR experiment. Shown are 2-kb upstream sequences of the *YUC* genes. The translational start site (ATG) is shown at position +1. (B) Gel photographs showing the amplified products from the ChIP assay. The ChIP assays were performed using 6-d-old seedlings expressing the PIF4-HA fusion protein untreated or treated with 29°C for 6 h. Antibody to the HA tag was used to immunoprecipitate PIF4-HA and associated DNA fragments. DNA was amplified by using primers specific to the region containing the G-box element or control regions in *ACT2* promoter as indicated. Shown are representative data from one biological replicate; this experiment was conducted for three biological replicates, yielding similar results. (C) EMSA assay showing that PIF4 binds the G-box motifs present in the *YUC8* promoter *in vitro*. The *YUC8* promoter fragments containing the G-box motifs were incubated with *in vitro* TNT-expressed PIF4 protein as indicated. Competition for PIF4 binding was performed with 10×, 20× and 50× cold *YUC8* probes containing G-box (G-wt, CACGTG) or mutated G-box (G-mut, CACGGG), respectively. FP, free probe. TnT indicates *in vitro*-expressed luciferase proteins used as a control.

Three lines of evidence support a scenario that the PIF4 transcription factor may directly regulate *YUC8* expression during high temperature-induced adaptation growth. First, underlying high temperature-induced hypocotyl elongation, high temperature upregulates the expression of *PIF4* in a similar fasion to that of *YUC8*. Second, high temperature-induced upregulation of *YUC8* expression requires the function of PIF4. Third, overexpression of *PIF4* leads to increased expression of *YUC8* and elevated free IAA levels *in planta*. Given that PIF4 specifically binds to a core DNA G-box motif (CACGTG) of its target gene promoters [18], we searched for the presence of G-box motifs in the promoter regions of the 11 *YUC* family genes present in the *Arabidopsis* genome. As shown in Figure 3A, G-box motifs were found not only in the promoter of *YUC8*, whose expression was significantly induced by high temperature (Figure 1), but also in the promoters of *YUC5*, *YUC9* and *YUC10*, whose expression was not or slightly induced by high temperature (Figure S2). To test the idea that PIF4 may actually bind to the G-box-containing regions of these *YUC* genes, we performed chromatin immuno-precipitaiton (ChIP) assays using a previously reported transgenic line expressing a fusion of PIF4 to the haemagglutinin (HA) antigen (PIF4-HA) [19] and anti-HA antibody (Abcam). PCR amplification of the promoter regions of the four *YUC* genes showed that PIF4-HA specifically bound to the G-box-containing promoter region of *YUC8*, but not to the G-box-containing promoter regions of *YUC5*, *YUC9* and *YUC10* (Figure 3B). These results suggest that PIF4 associates with the G-box DNA motifs in the promoter region of *YUC8 in vivo*. Further evidence supporting this conclusion came from electrophoretic mobility-shift assays (EMSA) using PIF4 protein expressed *in vitro*. As shown in Figure 3C, PIF4 bound to the G-box-containing DNA fragments present in the promoter region of *YUC8* and, this binding could be effectively competed by the addition of excess amount of unlabeled G-box-containing DNA probes (Figure 3C). As a control, we showed that DNA probes containing a mutated G-box motif (CACGGG) failed to compete the binding of PIF4 to the G-box-containing DNA fragments (Figure 3C). Together, these results support that the PIF4 transcription factor regulates *YUC8* expression by directly binding to its promoter region.

### PIF4 Activates *YUC8* Expression in the Transient Expression Assay

**Figure 4:**
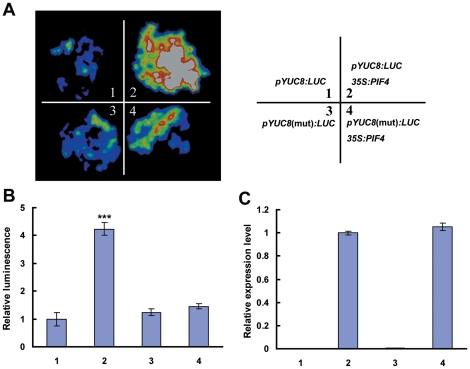
PIF4 Activates *YUC8* Expression, as Revealed by Transient Assays of *N. benthamiana* Leaves. (A) Transient expression assays showing that PIF4 activates the expression of *YUC8*. Representative images of *N. benthamiana* leaves 72 h after infiltration are shown. The right panel indicates the infiltrated constructs. (B) Quantitative analysis of luminescence intensity in (A). Five independent determinations were assessed. Error bars represent SD. Asterisks denote Student's *t*-test significance compared with control plants: ***, P<0.001. (C) qRT-PCR analysis of *PIF4* expression in the infiltrated leaf areas shown in (A). Total RNAs were extracted from leaves of *N. benthamiana* infiltrated with the constructs. Five independent determinations were assessed. Error bars represent SD.

Next, using the well-established transient expression assay of *Nicotiana benthamiana* leaves, we verified the activation effect of PIF4 on the expression of a reporter containing the *YUC8* promoter fused with the firefly luciferase (*LUC*) gene. When the *pYUC8:LUC* reporter was infiltrated into *N. benthamiana*, the LUC activity could be detected at lower level (Figure 4A, B). Coexpression of *pYUC8:LUC* with the *35S:PIF4* construct led to an obvious induction in luminescence intensity (Figure 4A, 4B), suggesting that ectopic expression of PIF4 can activate *pYUC8:LUC* expression in this transient expression assay. In a parallel experiment, *pYUC8*(mut)*:LUC*, in which the two G-boxes of the *YUC8* promoter were deleted and fused with *LUC*, togehter with *35S:PIF4* were co-infiltrated into *N. benthamiana* leaves. As shown in Figure 4, the activation effect of PIF4 on *pYUC8*(mut)*:LUC* expression was abolished. Together, our transient expression assays in *N. benthamiana* leaves confirmed that PIF4 directly activates *YUC8* expression *in vivo*.

### The *YUC8* Gene Is Required for PIF4–Mediated Hypocotyl Elongation

**Figure 5:**
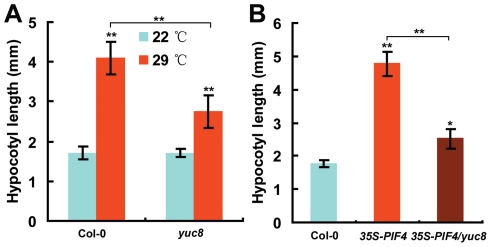
The *yuc8* Mutation Reduces the Induction of Hypocotyl Elongation by High Temperature and *PIF4* Overexpression. (A) Hypocotyl length showing that the *yuc8* mutation reduces high temperature-induced hypocotyl elongation. Four-d-old seedlings grown at 22°C were transferred to 29°C in continuous light for additional 2 d before hypocotyl length measurement. Data shown are average±SD. Asterisks represent Student's *t*-test significance between 29°C and 22°C grown plants for each genotype or between pairs indicated with brackets (**, P<0.01). Shown are representative data from one biological replicate; three biological replicates were conducted, yielding similar results. (B) Hypocotyl length showing that the *yuc8* mutation partially suppresses the long-hypocotyl phenotype of *35S-PIF4* plants. The hypocotyl length of 6-d-old seedlings of the indicated genotypes grown at 22°C was measured. Data shown are average±SD. Asterisks represent Student's *t*-test significance between transgenic/mutant and wild-type plants or between pairs indicated with brackets (*, P<0.05; **, P<0.01). Shown are representative data from one biological replicate; three biological replicates were conducted, yielding similar results.

To determine the genetic relationship between *PIF4* and *YUC8*, we identified a *yuc8* mutant (SALK_096110) which harbors a T-DNA insertion that markedly reduced the expression levels of the *YUC8* gene (Figure S7). We show that the *yuc8* mutant is defective in high temperature-induced hypocotyl growth (Figure 5A). We then introduced the above-described *35S-PIF4* construct into the genetic background of the *yuc8* mutant through genetic crossing. As shown in Figure 5B, the *yuc8* mutation substantially suppressed the long-hypocotyl phenotype of the *35S-PIF4* plants, supporting that *YUC8* acts genetically downstream of *PIF4* in regulating high temperature-induced hypocotyl elongation.

### SHY2/IAA3 Is an Important Component of the PIF4–Auxin Pathway in Regulating High Temperature–Induced Hypocotyl Elongation

**Figure 6:**
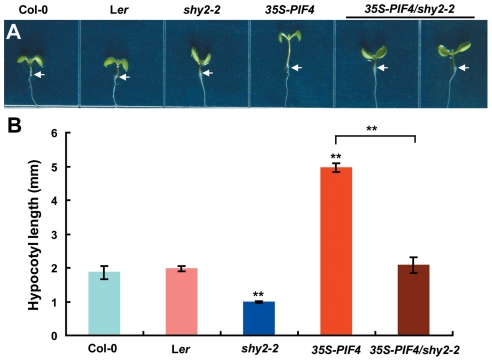
The *shy2-2* Mutation Suppresses the Long-Hypocotyl Phenotype of *35S-PIF4* Seedlings. (A) Representative images showing that *shy2-2* suppresses the long-hypocotyl phenotype of *35S-PIF4*. Shown are 6-d-old seedlings of Col-0, L*er*, *shy2-2*, *35S-PIF4* and *35S-PIF4/shy2-2* grown at 22°C. (B) Hypocotyl length showing that *shy2-2* suppresses the long-hypocotyl phenotype of *35S-PIF4*. Hypocotyl length of six-d-old seedlings of the indicated genotypes grown at 22°C was measured. Data shown are average±SD. Student's *t*-test between mutant/transgenic and wild-type seedlings was performed (**, P<0.01). Shown are representative data from one biological replicate; three biological replicates were conducted, yielding similar results.

Several elegant observations have demonstrated the involvement of PIF4 and auxin in regulating adaptive growth of plants to high temperature [1], [16]. Our data presented here further revealed that, through directly activating of the *YUC8* expression, PIF4 integrates its action into the auxin pathway in regulating high temperature-mediated hypocotyl elongation. To further identify auxin signaling components involved in this process, we employed a genetic approach to search for auxin-related mutations that can suppress the long-hypocotyl phenotype of the *35S-PIF4* plants. It has been shown that the *shy2-2* mutant, which harbors a stabilized mutant form of the SHY2/IAA3 protein, displays a short hypocotyl phenotype [20], suggesting a role of SHY2/IAA3 in regulating auxin-mediated hypocotyl growth. We showed that *shy2-2* seedlings are defective in high temperature-induced hypocotyl growth (Figure S3). Importantly, like the auxin signaling mutant *axr1-12* (Figure S8), *shy2-2* genetically suppressed the long-hypocotyl phenotype of *35S-PIF4* (Figure 6). In contrast, other gain-of-function mutations in different IAA proteins [21]–[24], including *slr-1* (contains a gain-of-function mutation in IAA14), *axr2-1* (contains a gain-of-function mutation in IAA7), *axr5-1* (contains a gain-of-function mutation in IAA1) and *iaa28-1*, did not affect hypocotyl elongation in response to high temperature and failed to suppress the long-hypocotyl phenotype of *35S-PIF4* seedlings (Figure S9). These results demonstrate that the auxin signaling repressor SHY2/IAA3 is selectively involved in high temperature-induced hypocotyl growth.

## Discussion

As sessile organisms, plants have evolved remarkable ability to adapt their development to the ever-changing environmental conditions. Exposure of plants to high temperature results in dramatic changes in plant architecture, including elongation responses and leaf hyponasty. High temperatures can also considerably reduce plant biomass, raising concerns over future crop productivity and food security. Therefore, the modulation of plant architecture by high temperature is a subject of considerable agricultural significance, particularly with regard to global climate change. An ever-growing body of evidence in *Arabidopsis* has implicated that high temperature-induced plant architecture remodeling relies on the interplays between multiple external and internal cues including light, circadian clock, auxin, gibberellin and others [25], [26]. Particularly, recent studies reveal that a group of bHLH transcription factors play a central role in modulating developmental responses to both light and temperature [1], [14], [16], [27]–[29].

### PIF4 Is an Integrator between High Temperature and Auxin Pathway in Regulating Adaptive Hypocotyl Growth

In this study, we discovered that, as a molecular integrator, the PIF4 transcription factor links high temperature to the auxin pathway in regulating high temperature-induced hypocotyl elongation. Several lines of evidence support this finding: First, underlying the long-standing observation that high temperature induces a dramatic elongation of the hypocotyl, we showed that high temperature triggers an elevation of the transcript abundance of both *PIF4* and *YUC8* (Figure 1). Second, high temperature-induced upregulation of *YUC8* expression largely depends on the function of PIF4 (Figure 1). Third, overexpression of *PIF4* leads to increased expression of *YUC8* and elevated endogenous free IAA levels (Figure 2). Fourth, as revealed by ChIP and EMSA assays, PIF4 specifically binds to a core DNA G-box motif (CACGTG) present in the promoter of the *YUC8* gene (Figure 3). Fifth, transactivation assays in *N. benthamiana* leaves support that PIF4 stimulates the activity of the *YUC8* promoter fused with a reporter (Figure 4). Finally, the *yuc8* mutation, which is defective in high temperature-induced hypocotyl elongation, is able to partially suppress the long-hypocotyl phenotype of the *35S-PIF4* plants (Figure 5). Together, these data support that, PIF4 selectively activates the expression of the auxin biosynthetic gene *YUC8*, thus integrates high temperature to the auxin pathway in regulating adaptive hypocotyl growth.

It is worthy of note that the *yuc8* mutant still retains some response to high temperature in hypocotyl elongation and that this mutation fails to completely suppress the long-hypocotyl phenotype of *35S-PIF4* plants (Figure 5). A plausible explanation for this is that the *yuc8* mutant used in this study shows reduced, but not loss of, *YUC8* expression (Figure S7). Alternatively, we could not rule out the possibility that PIF4 may activate auxin biosynthetic genes other than *YUC8*, which act weakly in PIF4-mediated hypocotyl growth in response to high temperature. A very recent report hints that the PIF4 transcription factor could target *TAA1* [29], which acts genetically upstream of the *YUC* family genes in IAA production [12], [13]. Considering that overexpression of *TAA1* does not lead to any obvious developmental phenotype [11], [12] and that *TAA1* and *YUCs* act in a common linear biosynthetic pathway for auxin production [6], [12], [13], it is reasonable to propose that *TAA1* acts together with other auxin biosynthesis genes such as *YUC8* to mediate high temperature-induced and PIF4-mediated hypocotyl elongation. However, our gene expression analyses reveal that overexpression of *PIF4* alone fails to elevate *TAA1* transcription (Figure S4).

PIF4-mediated activation of *YUC8* expression in response to high temperature exemplifies a mechanism by which environmental cues manipulate auxin, the key endogenous modulator of plant architecture. Another known physiological process in which both PIF4 and auxin are involved is shade avoidance syndrome (SAS), plant adaptive growth responses to the light signal [14], [30], [31]. PIF4 is therefore emerging as a molecular “hub” to integrate both temperature and light signals to regulate plant architecture remodeling [14]. Accumulating evidence reveals that, unlike shade avoidance, where PIF4 acts redundantly with its homolog, PIF5, to regulate elongation growth, PIF4 appears to perform a dominant role in driving high temperature-induced adaptive growth [1], [14], [16], [32]–[34]. These studies suggest that, PIF4, and possibly other PIF family members, have specialized and overlapping functions in regulating plant adaptive growth to different environmental stimuli.

### SHY2/IAA3 Is Specifically Involved in PIF4–Mediated Hypocotyl Elongation in Response to High Temperature

Our results support a scenario in which the auxin pathway acts downstream of the PIF4 transcriptional regulator in regulating high temperature-induced hypocotyl elongation. Supporting evidence for this hypothesis came from our genetic analysis showing that the *axr1-12* mutation, which contains a mutation in a subunit of the heterodimeric RUB-E1 enzyme required for auxin signaling [35], completely suppressed the long-hypocotyl phenotype of *35S-PIF4* seedlings (Figure S8). Based on our current knowledge of the auxin signaling pathway, auxin mediates the expression of auxin responsive genes through the inactivation of AUX/IAA transcriptional repressors that negatively control the activity of AUXIN REPONSE FACTOR (ARF) transcription factors [36]. In the context that many gain-of-function *aux/iaa* mutations are associated with reduced response to exogenous auxin, but developmental defects among these mutants are frequently more specific [36], it is reasonable to speculate that specific Aux/IAA-ARF pair(s) may function in the PIF4-auxin pathway to mediate the specialized hypocotyl elongation process triggered by high temperature. In our genetic efforts to identify new components involved in the PIF4-auxin pathway in regulating high temperature-mediated hypocotyl elongation, we determined that SHY2/IAA3, but not other IAA proteins tested, has a specialized function in mediating high temperature-induced hypocotyl elongation. It is of interest in future studies to identify the ARF transcription factor(s) interacting with SHY2/IAA3 in regulating high temperature-induced hypocotyl elongation.

## Materials and Methods

### Plant Materials and Growth Conditions

*Arabidopsis thaliana* ecotypes Columbia (Col-0), L*er* and WS were used as wild types. The *pif4* mutant used in this study was the reported null allele *pif4-2* [1]. Other plant materials used in this study were previously described: *DR5:GUS* [37], *35S-PIF4* [19], *35S:PIF4-HA* [19], *yucca* [7], *axr1-12* [38], *shy2-2* [2019], *slr-1* [21], *axr2-1* [22], *axr5-1* [23] and *iaa28-1* [24]. *yuc8* (SALK_096110) was identified from the SIGnAL T-DNA collection [39].

All molecular manipulations were performed according to standard methods [40]. The *PIF4* coding fragment was amplified by PCR and cloned into the *Asc*I/*Pac*I sites of the binary vector *pMDC7* [17], resulting in a chemical-inducible *PIF4* expression construct. The construct was then transformed into *Agrobacterium tumefaciens* strain GV3101 (pMP90), which was used for transformation of *Arabidopsis* plants by vacuum infiltration [41].

Seeds were surface-sterilized for 15 min in 10% bleach, washed four times with sterile water, and plated on half-strength Murashige and Skoog (MS) medium. Plants were stratified at 4°C for 2 d in darkness and then transferred to a phytotrone set at 22°C with a 16-h light/8-h dark photoperiod or in continuous light for specific experiments. For high temperature treatment, plants were directly grown at 29°C in continuous light or young seedlings were transferred to 29°C in continuous light for different times.

### Gene Expression Analysis

For qRT-PCR analysis, seedling were harvested and frozen in liquid nitrogen for RNA extraction. RNA extraction and qRT-PCR analysis were performed as previously described [37]. Primers used to quantify gene expression levels are listed in Table S1. The GUS activity assays were performed as previously described [37].

### ChIP–PCR Assay

One gram of 6-d-old seedlings of *35S:PIF4-HA* transgenic plants [19] and the anti-HA antibody (Abcam) were used in ChIP experiments. Chromatin immunoprecipitation (ChIP) assays were performed as previously described [42]. The enrichment of DNA fragments was determined by semi-quantitative PCR analysis. Three independent biological repeats were performed.

### DNA Gel-Shift Assay

PIF4 and Luciferase (Luc) were synthesized by using the Rabbit Reticulocyte TNT system (Promega) [18], [43]. The 60-bp *YUC8* promoter probes containing G-box motifs were synthesized and labeled with biotin at the 3′ end (Invitrogen). Cold competitor probes were generated from dimerized oligos of the *YUC8* promoter region containing the wt-G-box (CACGTG) or mut-G-box (CACGGG) motifs, respectively. DNA gel-shift assays were performed as described [18], [43]. Probe sequences are shown in Table S1.

### Transactivation of *YUC8* Promoter Activity by PIF4 in *N. benthamiana* Leaves

The transient expression assays were performed in *N. benthamiana* leaves as previously described [44]. The *YUC8* promoter was amplified with the primer pairs 5-CACCATCCGATATGATAACGAT-3 and 5-TGGAAGTTGTATTGGAAA-3 and cloned into *pENTR* using the pENTR Directional TOPO cloning kit (Invitrogen). To generate *YUC8* promoter with mutations, site-directed mutagenesis was used to delete the two G-boxes in the *YUC8* promoter (Figure 3) using the TaKaRa MutanBEST kit. Then, the two *YUC8* promoter versions were fused with the luciferase reporter gene *LUC* through the Gateway reactions into the plant binary vector *pGWB35* [45] to generate the reporter constructs *pYUC8:LUC* and *pYUC8*(mut)*:LUC*. The PIF4 effector construct was the *35S:PIF4*. For this construct, the *PIF4* coding fragment was amplified by PCR with the primer pairs 5-CACCATGGAACACCAAGGTTGGAG-3 and 5-GTGGTCCAAACGAGAACCGT-3. Five independent determinations were assessed. Error bars represent SD. The experiments were repeated at least five times with similar results.

### Free IAA Measurement

For measurement of free IAA levels in wild-type and *pif4* mutant hypocotyls in response to high temperature treatment, the hypocotyls of 6-d-old wild-type and *pif4* mutant seedlings grown at 22°C and 29°C in continuous light, respectively, were harvested for free IAA measurement. For the wild-type seedlings grown at 29°C, the 2 mm length parts for each hypocotyl (above the junction between hypocotyl and root) were harvested for free IAA measurement. Eight-d-old seedlings of wild-type and *35S-PIF4* grown at 22°C in continuous light were harvested for free IAA measurement. Approximately 200 mg (fresh weight) of tissues were used for IAA extraction and measurement as previously described [46].

## Supporting Information

Figure S1 Comparison of *PIF4* and *YUC8* Expression in Response to High Temperature Treatment. (A–B) qRT-PCR analyses of the expression of *PIF4* and *YUC8* genes in wild type (Col-0) upon high temperature treatment. Six-d-old Col-0 seedlings grown at 22°C were transferred to 29°C in continuous light or were continually placed at 22°C for a time course, respectively. The 22°C and 29°C grown seedlings for each time point were harvested at the same time for RNA extraction and qRT-PCR analyses. The transcript levels of target genes were normalized to the *ACTIN7* expression and were relative to those of untreated seedlings (0 h). Data shown are average and SD of triplicate reactions. Shown are representative data from one biological replicate; three biological replicates were conducted, yielding similar results.
(TIF)

Figure S2 High Temperature–Induced Expression Patterns of *YUCs* in Wild Type (Col-0) and the *pif4* Mutant. (A–E) qRT-PCR analyses of the expression of *YUC* genes in wild type (Col-0) and the *pif4* mutant. Six-d-old Col-0 and *pif4* seedlings grown at 22°C were transferred to 29°C in continuous light or were continually placed at 22°C for a 24 h time course, respectively. The 22°C and 29°C grown seedlings for each time point were harvested at the same time for RNA extraction and qRT-PCR analyses. The transcript levels of target genes were normalized to the *ACTIN7* expression and were relative to those of untreated seedlings (0 h) for each genotype. Data shown are average and SD of triplicate reactions. Shown are representative data from one biological replicate; three biological replicates were conducted, yielding similar results.
(TIF)

Figure S3 High Temperature–Induced Adaptation Growth of Wild Type and Mutants. (A) Representative images showing high temperature-induced hypocotyl elongation of the indicated genotypes. Four-d-old seedlings grown at 22°C were transferred to 29°C for additional 2 d before photographs were taken. (B) Measurements of hypocotyl length of seedlings shown in (A). Four-d-old seedlings grown at 22°C were transferred to 29°C for additional 2 d before hypocoyl lengths were measured. Data shown are average±SD. Asterisks represent Student's *t*-test significance between 29°C and 22°C grown plants or between transgenic/mutant lines and their wild types (**, P<0.01). Shown are representative data from one biological replicate; three biological replicates were conducted, yielding similar results. (C) Photographs of the indicated genotypes grown in soil at different temperatures. Plants were grown at 22°C for 10 d before transfer to 29°C for additional 12 d. Control plants were maintained at 22°C.
(TIF)

Figure S4 Expression Analysis of *TAA1* in Wild Type (Col-0) and *35S-PIF4* Plants. Six-d-old Col-0 and *35S-PIF4* seedlings grown in normal growth conditions (22°C) were harvested at the same time for RNA extraction and qRT-PCR analyses. Transcript levels of *YUC8* were normalized to the *ACTIN7* expression and then were relative to those of Col-0 seedlings. Data shown are average and SD of triplicate reactions. Shown are representative data from one biological replicate; three biological replicates were conducted, yielding similar results.
(TIF)

Figure S5 Hypocotyl Growth Phenotype of *pMDC7:PIF4* Transgenic Line. Six-d-old seedlings of *pMDC7:PIF4* grown at 22°C on medium without or with inducer (10 µM estradiol).
(TIF)

Figure S6 Tissue-Specific Expression of *DR5:GUS* in WT and *35S-PIF4* Plants. The 6-day-old *DR5:GUS* and *DR5:GUS/35S-PIF4* seedlings grown at 22°C were used for GUS activity assays. Shown are representative photographs for basal region of hypocotyls from one biological replicate; three biological replicates were conducted, yielding similar results.
(TIF)

Figure S7 Molecular Analysis of the *yuc8* Mutant. (A) Diagram showing the T-DNA insertion site in the *YUC8* gene. (B) RT-PCR analysis showing reduced expression of *YUC8* in the *yuc8* mutant. (C) qRT-PCR analysis showing reduced expression of *YUC8* in the *yuc8* mutant. The transcript levels of *YUC8* were normalized to the *ACTIN7* expression and were relative to those of Col-0 seedlings. Data shown are average and SD of triplicate reactions. Student's *t*-test between Col-0 and *yuc8* plants was performed (**, P<0.01). Shown are representative data from one biological replicate; three biological replicates were conducted, yielding similar results.
(TIF)

Figure S8 The *axr1-12* Mutation Suppresses the Constant Long-Hypocotyl Phenotype of *35S-PIF4*. (A) Representative photographs of 6-d-old seedlings of the indicated genotypes grown at 22°C. Shown are representative data from one biological replicate; three biological replicates were conducted, yielding similar results. (B) Hypocotyl length measurements of seedlings shown in (A). Data shown are average±SD. Student's *t*-test between mutant/transgenic lines and wild type was performed (**, P<0.01). Shown are representative data from one biological replicate; three biological replicates were conducted, yielding similar results.
(TIF)

Figure S9 The Gain-of-Function Mutations *slr-1*, *axr2-1*, *axr5-1* and *iaa28-1* Do Not Affect High Temperature-Mediated Hypocotyl Elongation. (A) *slr-1*, *axr2-1*, *axr5-1* and *iaa28-1* display normal hypocotyl elongation in response to high temperature. Four-d-old seedlings of the indicated genotypes grown at 22°C were transferred to 29°C for additional 2 d before hypocoyl length measurement. Data shown are average±SD. Student's *t*-test between 29°C-treated and untreated plants was performed (**, P<0.01). Shown are representative data from one biological replicate; three biological replicates were conducted, yielding similar results. (B) *slr-1* and *axr5-1* mutations fail to suppress the long-hypocotyl phenotype of *35S-PIF4* seedlings. The hypocotyl lengths of 6-d-old seedlings of the indicated genotypes grown at 22°C were measured. Data shown are average±SD. Student's *t*-test between mutant/transgenic lines and wild type was performed (**, P<0.01). Shown are representative data from one biological replicate; three biological replicates were conducted, yielding similar results.
(TIF)

Table S1 List of the primers used in this study.
(DOC)
