# Supplementary figures and images for: PIF4–Mediated Activation of YUCCA8 Expression Integrates Temperature into the Auxin Pathway in Regulating Arabidopsis Hypocotyl Growth
Source: PLoS Genet. 2012 Mar 29;8(3):e1002594. doi: 10.1371/journal.pgen.1002594 (PMC3315464; doi:10.1371/journal.pgen.1002594)

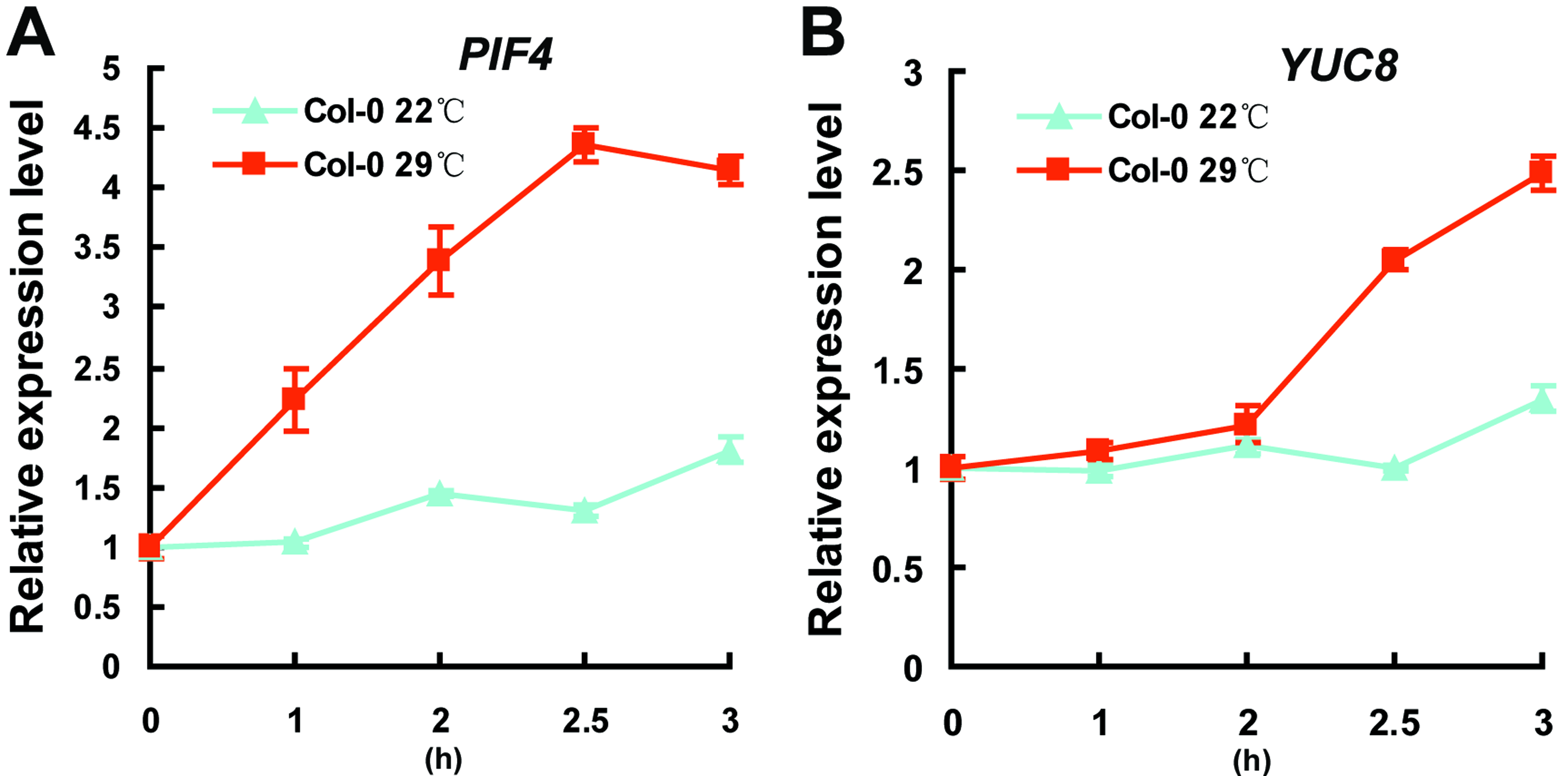

Supplement: Figure S1 — Comparison of PIF4 and YUC8 Expression in Response to High Temperature Treatment. (A–B) qRT-PCR analyses of the expression of PIF4 and YUC8 genes in wild type (Col-0) upon high temperature treatment. Six-d-old Col-0 seedlings grown at 22°C were transferred to 29°C in continuous light or were continually placed at 22°C for a time course, respectively. The 22°C and 29°C grown seedlings for each time point were harvested at the same time for RNA extraction and qRT-PCR analyses. The transcript levels of target genes were normalized to the ACTIN7 expression and were relative to those of untreated seedlings (0 h). Data shown are average and SD of triplicate reactions. Shown are representative data from one biological replicate; three biological replicates were conducted, yielding similar results. (TIF) [file pgen.1002594.s001.tif]

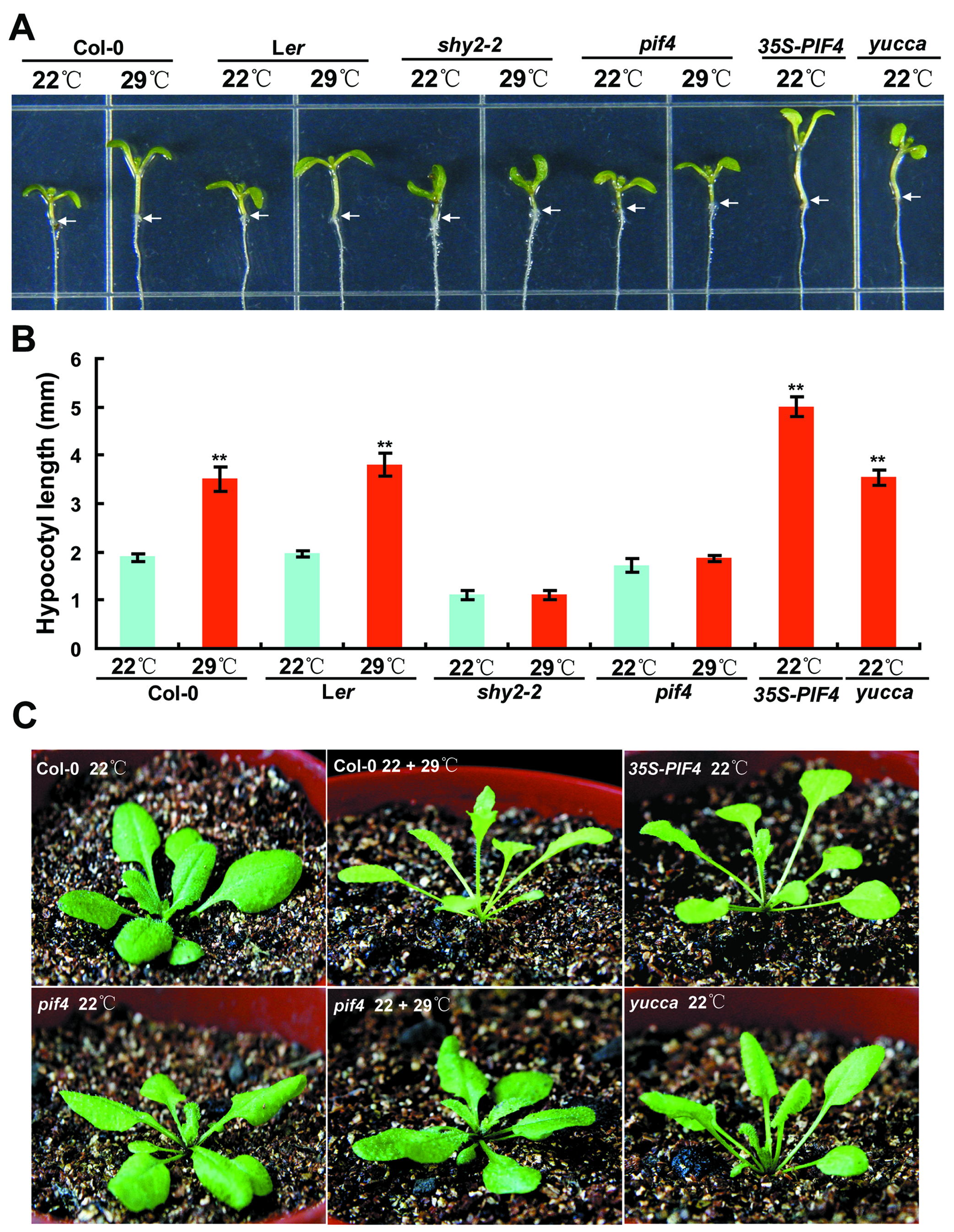

Supplement: Figure S3 — High Temperature–Induced Adaptation Growth of Wild Type and Mutants. (A) Representative images showing high temperature-induced hypocotyl elongation of the indicated genotypes. Four-d-old seedlings grown at 22°C were transferred to 29°C for additional 2 d before photographs were taken. (B) Measurements of hypocotyl length of seedlings shown in (A). Four-d-old seedlings grown at 22°C were transferred to 29°C for additional 2 d before hypocoyl lengths were measured. Data shown are average±SD. Asterisks represent Student's t-test significance between 29°C and 22°C grown plants or between transgenic/mutant lines and their wild types (**, P<0.01). Shown are representative data from one biological replicate; three biological replicates were conducted, yielding similar results. (C) Photographs of the indicated genotypes grown in soil at different temperatures. Plants were grown at 22°C for 10 d before transfer to 29°C for additional 12 d. Control plants were maintained at 22°C. (TIF) [file pgen.1002594.s003.tif]

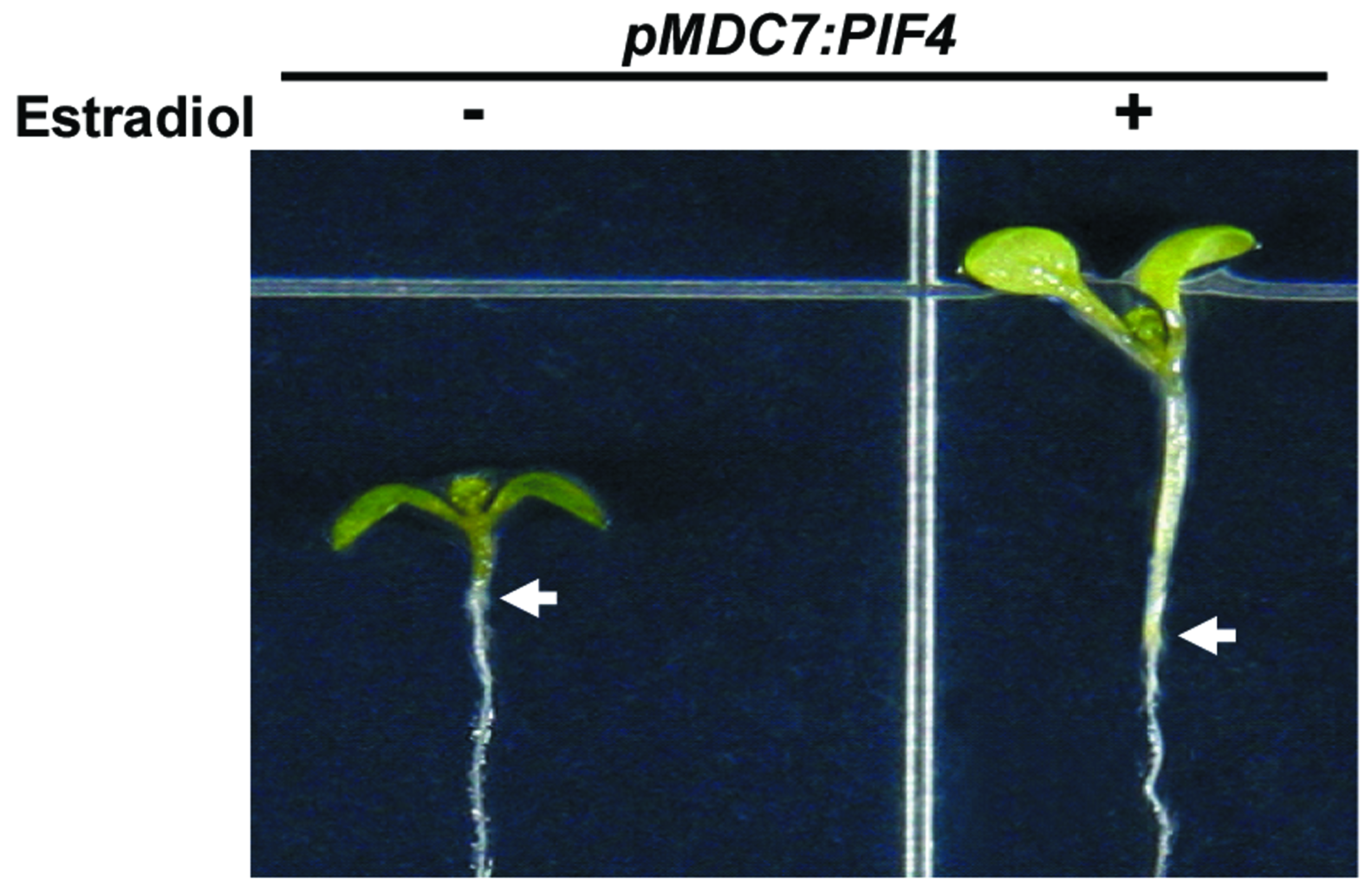

Supplement: Figure S5 — Hypocotyl Growth Phenotype of pMDC7:PIF4 Transgenic Line. Six-d-old seedlings of pMDC7:PIF4 grown at 22°C on medium without or with inducer (10 µM estradiol). (TIF) [file pgen.1002594.s005.tif]

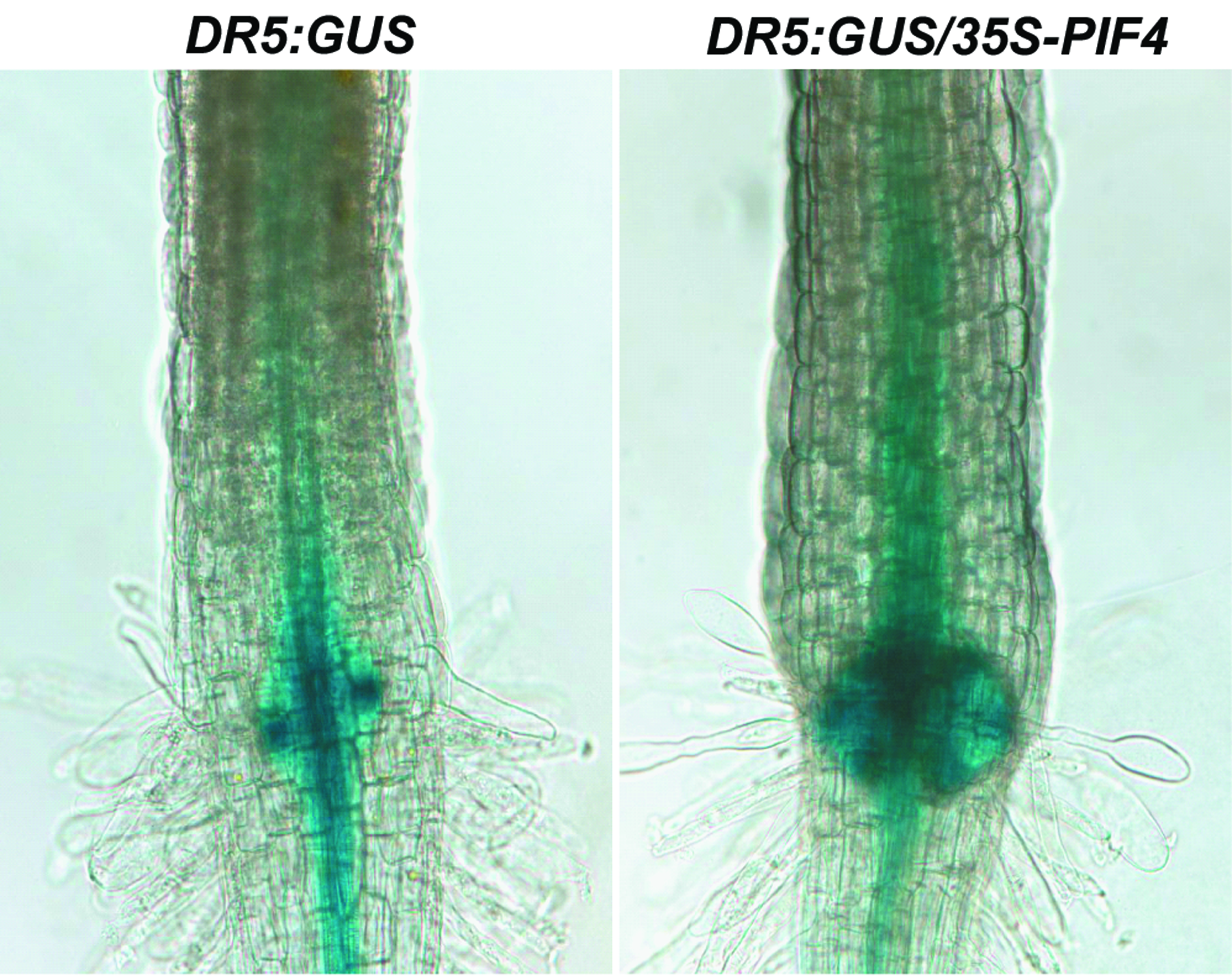

Supplement: Figure S6 — Tissue-Specific Expression of DR5:GUS in WT and 35S-PIF4 Plants. The 6-day-old DR5:GUS and DR5:GUS/35S-PIF4 seedlings grown at 22°C were used for GUS activity assays. Shown are representative photographs for basal region of hypocotyls from one biological replicate; three biological replicates were conducted, yielding similar results. (TIF) [file pgen.1002594.s006.tif]

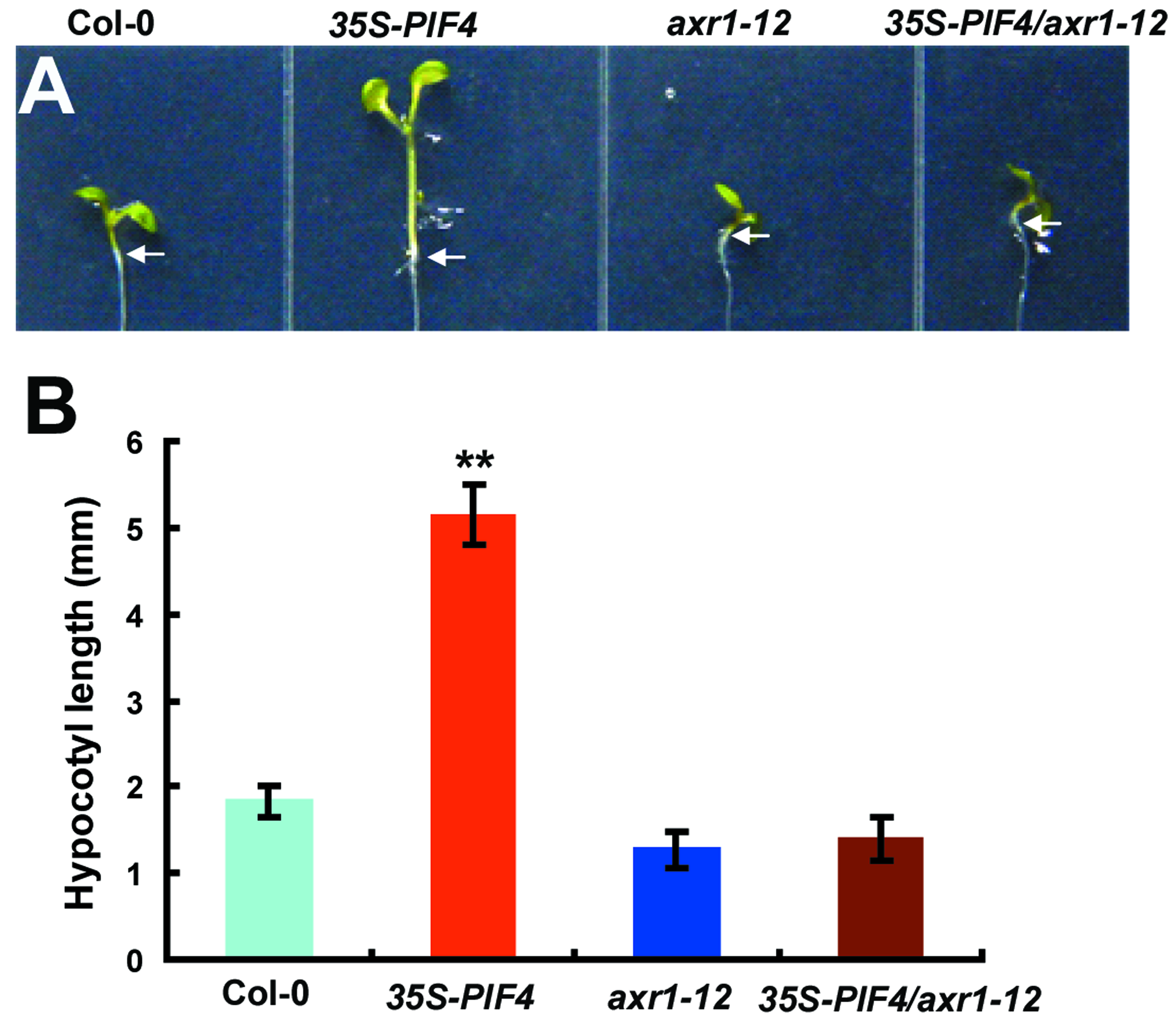

Supplement: Figure S8 — The axr1-12 Mutation Suppresses the Constant Long-Hypocotyl Phenotype of 35S-PIF4. (A) Representative photographs of 6-d-old seedlings of the indicated genotypes grown at 22°C. Shown are representative data from one biological replicate; three biological replicates were conducted, yielding similar results. (B) Hypocotyl length measurements of seedlings shown in (A). Data shown are average±SD. Student's t-test between mutant/transgenic lines and wild type was performed (**, P<0.01). Shown are representative data from one biological replicate; three biological replicates were conducted, yielding similar results. (TIF) [file pgen.1002594.s008.tif]
