# Supplementary material for: PIF4–Mediated Activation of YUCCA8 Expression Integrates Temperature into the Auxin Pathway in Regulating Arabidopsis Hypocotyl Growth
Source: PLoS Genet. 2012 Mar 29;8(3):e1002594. doi: 10.1371/journal.pgen.1002594 (PMC3315464; doi:10.1371/journal.pgen.1002594)
Supplement: Table S1 — List of the primers used in this study. (DOC) [file pgen.1002594.s010.doc]

**Table S1. List of the primers used in this study.**

| **Primer name** | **Sequence (5’ to 3’)** |
| --- | --- |
| **For qRT-PCR**  ACT7(q)F  ACT7(q)R  PIF4(q)F  PIF4(q)R  YUC1(q)F  YUC1(q)R  YUC2(q)F  YUC2(q)R  YUC5(q)F  YUC5(q)R  YUC8(q)F  YUC8(q)R  YUC9(q)F  YUC9(q)R  YUC10(q)F  YUC10(q)R  TAA1(q)F  TAA1(q)R  **For pMDC7:PIF4**  PIF4F  PIF4R  **For *yuc8* mutant**  YUC8(salk)LP YUC8(salk)RP  LBa  **For ChIP-PCR**  YUC8(ChIP)F  YUC8(ChIP)R  YUC5(ChIP)F  YUC5(ChIP)R  YUC9(ChIP)F  YUC9(ChIP)R  YUC10(ChIP)F  YUC10(ChIP)R  ACT2(ChIP)F  ACT2(ChIP)R  **For PIF4 translation**  ***in vitro***  PIF4(TNT)F  PIF4(TNT)R  **DNA-gel shift assay**  YUC8p probe | TCCATGAAACAACTTACAACTCCATCA  CATCGTACTCACTCTTTGAAATCCACA  CCAGATCATCTCCGACCGGTTTG  CTAGTGGTCCAAACGAGAACCGT  tggagagtaaagactcatgat  gtactcactcgcgtgaacgat  ggtgacacggatcggttagggt  tgccgaataatgcattacccgt  TTCAACGAGTGTGTCCAGTCTGCT  TCTCTGGAACAACTTTCTCCGCGT  TGTATGCGGTTGGGTTTACGAGGA  CCTTGAGCGTTTCGTGGGTTGTTT  CCTGCAATCAAACAGTTCTCGCGT  TGAAGCCAAGAAGGGACGTTGCTA  TTCTGAAGTATGCTCCAGTGGCGA  GTTTGGTGGCGAAAGGACCTTGTT  CCCTGCGTTTGCGTGGCTAGGGA  GAGCTTCATGTTGGCGAGTCTCT  gggGGCGCGCCATGGAACACCAAGGTTGGAGTTT  gggTTAATTAACTAGTGGTCCAAACGAGAACCGT  ATTCTGCATTTGGTTCCACAC  GACTCACTCTTCGACACGGTC  TGGTTCACGTAGTGGGCCATCG  gggaatgggtttgatgtggaatt  gagaagggaagtgatggaattag  TGGGCTCACCATCCTATC  GAAACCGAATGAACAACA  cccgctcttgactcatcatcacc  ccctccaccatatatgattgtta  gaccgtacatcgtctctatagtc  gtgatgcacatcaagaagaatgg  CGTTTCGCTTTCCTTAGTGTTAGCT  CACAACGCATGCTAAACAGATCTAG  ACAGAATTCATGGAACACCAAGGTTGGAG  ACAGTCGACCTAGTGGTCCAAACGAGAAC  ctcatcctctcCACGTGgcttccattccacgtcatctttttcttcccCACGTGgcttcctctcg |
